# Spatial and Spectral Dependencies of Maize Yield Estimation Using Remote Sensing

**DOI:** 10.3390/s24123958

**Published:** 2024-06-18

**Authors:** Nathan Burglewski, Subhashree Srinivasagan, Quirine Ketterings, Jan van Aardt

**Affiliations:** 1 Center for Imaging Science, Rochester Institute of Technology, Rochester, NY 14623, USA; jvacis@rit.edu; 2 Nutrient Management Spear Program, Cornell University, Ithaca, NY 14850, USA; sn558@cornell.edu (S.S.); qmk2@cornell.edu (Q.K.)

**Keywords:** imaging spectroscopy, hyperspectral imagery, agriculture, yield, vegetation, spectral indices, unmanned aerial systems, multispectral, resampling

## Abstract

Corn (*Zea mays* L.) is the most abundant food/feed crop, making accurate yield estimation a critical data point for monitoring global food production. Sensors with varying spatial/spectral configurations have been used to develop corn yield models from intra-field (0.1 m ground sample distance (GSD)) to regional scales (>250 m GSD). Understanding the spatial and spectral dependencies of these models is imperative to result interpretation, scaling, and deploying models. We leveraged high spatial resolution hyperspectral data collected with an unmanned aerial system mounted sensor (272 spectral bands from 0.4–1 μm at 0.063 m GSD) to estimate silage yield. We subjected our imagery to three band selection algorithms to quantitatively assess spectral reflectance features applicability to yield estimation. We then derived 11 spectral configurations, which were spatially resampled to multiple GSDs, and applied to a support vector regression (SVR) yield estimation model. Results indicate that accuracy degrades above 4 m GSD across all configurations, and a seven-band multispectral sensor which samples the red edge and multiple near-infrared bands resulted in higher accuracy in 90% of regression trials. These results bode well for our quest toward a definitive sensor definition for global corn yield modeling, with only temporal dependencies requiring additional investigation.

## 1. Introduction

Monitoring and assessment of crop production are critical components of tracking food security and economic stability globally. Governments, farmers, and non-government organizations (NGOs) all understandably have an interest in optimizing growing efficiency and maximizing yield. As such, many studies have investigated different methods of determining crop growth throughout the growing season, identifying and tracking yield drivers, nutrient uptake, and yield prediction and forecasting [1,2,3,4,5,6,7,8,9,10]. Remote sensing has a long history of aiding such precision agriculture efforts towards optimization of crop management decisions, while maximizing production, with corn grain and silage playing a central role due to its global ubiquity and being the most abundant food crop grown today [11,12,13,14,15].

The drivers of crop yield observable and exploitable by remote sensing applications include soil composition, nutrient availability, water quality and content, air and ground temperature (growing degree days [GDD]), plant density, plant height, sowing date, fraction of absorbed photosynthetically active radiation (fAPAR), leaf chlorophyll concentration/content (LCC), and carotenoid content (CC), among others [16,17,18,19]. Regression models for these yield drivers rely on the sensor’s sampling spectrum, applying this reflectance data to the calculation of vegetation indices (VIs) for correlation analysis. It is therefore worthwhile to investigate which spectral bands provide the best sensitivity for yield regression modeling to identify the appropriate band centers and widths for use in VI calculation.

Corn yield forecast and estimation models have been built upon and improved alongside progress made in remote sensing science. These models leverage the knowledge gained about remotely sensed yield drivers and the correlation of spectral content with yield. An investigation into these correlations reveals the relationships that VIs share and the biophysical and biochemical parameters for which they were derived. However, the models for these crop parameters, like LCC and CC, vary depending on corn variety, soil composition, local weather conditions, and management practices [20,21,22,23,24,25,26,27]. Many studies have found that these parameters can be fit with either a linear or exponential model, while recent progress has been made in engaging machine learning solutions [14,28,29,30]. Machine learning and artificial intelligence-based approaches to yield modeling have shown great promise in improving the accuracy of yield forecast and prediction models, while also revealing the flexibility that these models require in order to perform well in multiple environments [14,28,31,32]. The consideration of “scale”, i.e., the spatial unit size at which models were developed or will be implemented at, also has received considerable attention.

Corn yield forecast and prediction models are typically tailored to a single scale, which is designated with a particular set of goals in mind. Within-field early season estimations aim to inform management decisions, while county-level and larger models are used to gauge economic trends, food security, and other production-related metrics [6,29]. Within-field yield forecasts also can be used to identify low-yield zones within a farm, helping farmers plan long-term solutions which can make their farms more efficient [29,33]. Furthermore, harvester-mounted yield monitors require complex calibration procedures, while also being prone to human error during collection. Field-scale yield mapping seeks to map low and high-performance zones during the growing season can help inform farmers of more effective field management decisions [29,33]. It is in these contexts that remote sensing has played an increasingly important role for crop yield assessment.

Linear, multiple linear, and exponential regression are commonly used corn silage and grain yield forecast and prediction models. These models typically depend solely on remote sensing data and are fit to a VI, e.g., NDVI or EVI. Shajahan et al. (2021) used a UAS-mounted multispectral sensor for an exponential regression model to estimate intra-field grain yield throughout the growing season, reporting unreliable, low accuracy results before the VT, or tassel vegetative growth stage, but with increase to R^2^ = 0.94 when the plants reached full maturity [29]. Tunca et al. (2023) used satellite remote sensing data to fit a silage yield linear regression model which compared three different VIs over and assessed model accuracy throughout the growing season, resulting in R^2^ values ranging from 0.76 early in the growing season using the Simple Ratio VI to 0.98 using NDVI at the VT growth stage [34]. Tagarakis and Ketterings (2017) fit an exponential model to NDVI using data gathered using a handheld reflectance sensor, finding R^2^ = 0.82 for silage yield and R^2^ = 0.67 for grain yield [35]. Such regression approaches, however, recently have been augmented by relatively novel machine learning models. 

Machine learning algorithms used for yield forecasting and prediction modeling include the gradient boost method (GBM), extreme gradient boost method (xGBM), random forest (RF), and support vector regression (SVR). Barzin et al. (2022) examined the performance of these four algorithms for accuracy against one another using the same dataset, based on spectral reflectance data collected with a point spectrometer. The authors used both the entire spectral range, 400–1000 nm sampled at four wavelengths with unknown bandwidth as opposed to our 272-band imaging spectroscopy data, as well as VIs derived from the reflectance data as inputs to separate models for a further comparison. In all cases, SVR performed better than the other algorithms with an R^2^ = 0.76, while the VI dataset performed slightly better than the reflectance spectra [28]. Kenduiywo et al. (2020) leveraged USGS MODIS (36 spectral bands, 250 m GSD) data to compare SVR and RF models for 37 counties in Kenya across seven years. The authors found that SVR slightly outperformed the RF model with R^2^ = 0.70 and 0.69, respectively [14]. As SVR has been shown to perform well at both the sub-field and county scales when compared to other machine learning algorithms, we chose this method for our yield forecasting, thereby allowing us to focus on investigating the spatial and spectral resolution dependencies which may impact model performance. While these studies used multispectral reflectance measurements and imagery, it is worthwhile to more closely examine the relationship between model performance and spectral content through use of imaging spectroscopy data.

While these models use data which sample similar regions of the visible–near-infrared (VNIR) spectrum, they differ in band center location and bandwidths. Additionally, pixel pitch and optical design vary greatly depending on the platform mount and platform motion, thus generating a wide distribution of spatial resolutions, even between different implementations of the similar sensors [29,31,32]. 

These sensor characteristics have a distinct impact on data quality and reliability, and thus the calculation of a VI can change substantially from sensor to sensor, as was demonstrated by Fan and Liu (2016) in their investigation in sensor-based differences in NDVI measurements from satellite platforms [36]. This implies that an analysis of the spectral resolution dependencies of yield models which use these data is warranted, since slight changes in recorded reflectance values can lead to underperformance of a model fit to data from a different model sensor. 

Spatial resolution understandably plays a pivotal role in determining the scale of any yield model, but the variability of this resolution within any scale, i.e., using 3 m GSD imagery vs. 30 m GSD imagery for regional yield estimation, may have an influence on model performance due to aggregating the variability present within the data at any scale. Consider Deines et al. (2021), who investigated the scalable crop yield mapper (SCYM) using Landsat data (30 m GSD) for a nine-state-wide yield prediction and mapping study, while Sakamoto et al. (2013) used the United States Geological Survey’s Moderate-resolution Imaging Spectrometer (MODIS) (250 m GSD) to conduct a similar study over 34 states, resulting in RMSE = 0.78 Mg/ha and 1.39 Mg/ha, respectively [6,7]. While both studies investigated corn grain yield models, Aghighi et al. (2018) developed a machine learning sileage yield model using time-series Landsat imagery, testing boosted regression tree, support vector regression, random forest regression, and gaussian process regression, finding that boosted tree regression performed the best (RMSE = Mg/Ha) [37].

The corn yield models derived for each of these studies rely on time series data to predict corn grain or silage yield at the county and larger scale by estimating the growth stage throughout the season. Other studies, at the field level using unmanned aerial system (UAS) mounted sensors, gather data at centimeter scale GSDs. As the average leaf area of a corn plant at the end of the growth phase from a nadir facing sensor is approximately 0.55 m^2^ (unpublished data), it warrants further investigation into the optimum GSD for remote sensing data to be used for yield modeling. 

While these studies join the vast majority of corn yield modeling research which focus on deriving models for corn grain yield forecasting, corn is often harvested for silage for use in livestock feed and other corn products and these model parameters and accuracies differ substantially based upon the intended harvesting method. The United States Department of Agriculture (USDA) reports that in 2023, our study area, New York, USA, grew 242 thousand hectares of grain corn and 174 thousand hectares of silage corn [37]. It is therefore worthwhile to investigate the applicability to gains made in corn grain estimation modeling to corn silage yield modeling. 

Our study has two objectives: first, we test the applicability of a SVR corn yield model on silage corn as opposed to grain only corn, as SVR has been shown to out-perform other machine learning regression algorithms at multiple scales for grain yield estimation. Secondly, we sought to ascertain the spatial and spectral dependencies of such a model, leveraging our high dimensional data and advances made in hyperspectral band selection. The ultimate goal of this work was to identify a spectral sampling schema and regression model which is robust to scaling, i.e., an approach that performs well at smaller GSDs for field-level yield estimation, as well as at larger GSDs for regional yield estimation. This had to be accomplished while avoiding high data dimensionality through the mature field of spectral dimensional reduction, in an effort to reduce computational complexity. It is perhaps worth noting that our intention was not to benchmark different machine learning approaches, since a body of literature exists on this topic, e.g., Kenduiywo et al. (2020) and Barzin et al. (2022) [14,28]. The key focus was to assess the interplay between spectral and spatial system properties for corn yield assessment.

## 2. Materials and Methods

### 2.1. Study Area

**Figure 1:**
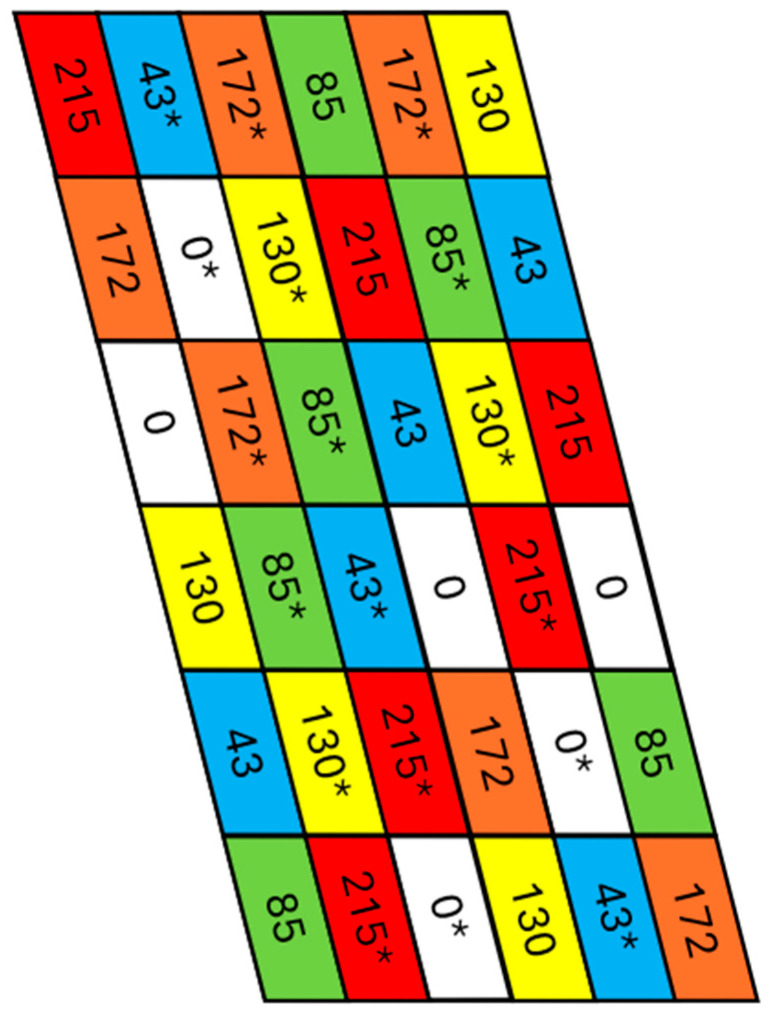
The layout of the study area. Each subplot is 15 × 61 m, and the numerical labels correspond to the nitrogen fertilization rate that a plot received (kg/ha) at sidedress time. An asterisk denotes a subplot which received manure at the beginning of the growing season.

The field used for this study is located in central New York, United States. The selected field was chosen due to its participation in a nitrogen rate and manure value analysis study, which consisted of six different nitrogen fertilization rates: 0, 43, 85, 130, 172, or 215 kg/ha. Each subplot was 15 × 61 m, and the entire field area was approximately 3.34 hectares, divided into 36 different subplots (Figure 1), and half of these subplots received manure from a tractor drawn manure spreader prior to planting.

Half of these subplots received manure at the beginning of the growing season while the other half received only fertilization treatment. Sidedressing (fertilization) took place 33 days after sowing (DAS) when corn was at the V5 growth stage. The V5 growth stage is also typically when the growing point of the plant emerges from the soil, which signifies the final opportunity to apply fertilizers at maximum efficiency, while avoiding damage to crops during application [38]. This field was harvested for silage, meaning that the entire plant was collected, as opposed to a grain harvest, where only the ear of corn is collected. 

### 2.2. Yield Data

Silage yield data were gathered during harvest using a chopper-mounted yield monitor system, consisting of a mass flow sensor, a moisture sensor, and a GPS unit. This information was then converted to an estimation of yield [39].

Yield data were cleaned using the procedure outlined by Kharel et al. (2019), the relative spatial consistency of all the yield datapoints was assessed to filter harvester pass overlaps and flow/moisture measurement delays using the supplied GPS data [33].

### 2.3. Data Collection

**Figure 2:**
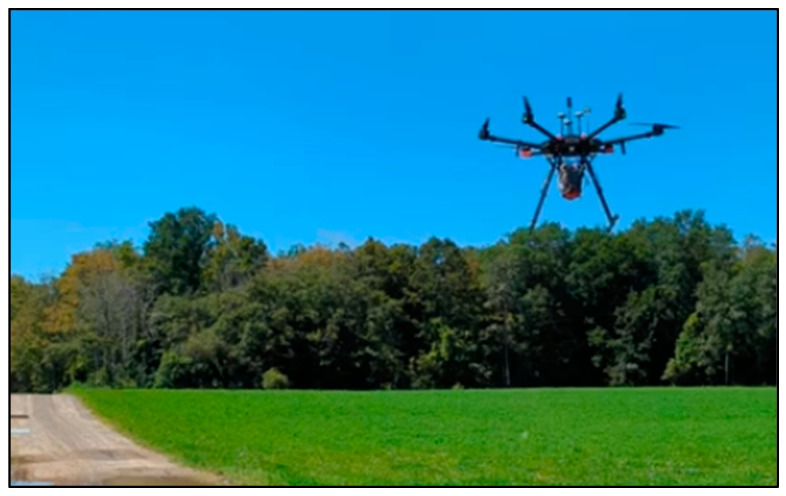
The UAS imagery collection platform used for this study, a DJI Matrice 600 Hexacopter. On board is a 272-band imaging spectrometer, a five-band multispectral imaging sensor, LIDAR, thermal imaging sensor, high resolution RGB imaging sensor, a downwelling spectrometer, and GPS navigation equipment.

Spectroscopic imagery of the study area was collected 79 DAS, when most corn was at the V-Tassel (VT) to R1 growth stage. The UAS-mounted sensor suite used included a multi-spectral sensor (RedEdge-5, MicaSense, Wichita, KS, USA), a high-resolution RGB imaging sensor (Mako G419, Allied Vision, Stadtroda, Germany), a VNIR imaging spectrometer with 272 bands, ranging from 400–1000 nm (Headwall Nano Hyperspec VNIR, Headwall Photonics, Bolton, MA, USA), and a SWIR imaging spectrometer with 170 bands, ranging from 1000–2500 nm (Headwall Nano Hyperspec SWIR) (Figure 2).

The VNIR spectroscopic imagery values for each pixel are in units of radiance (W × cm^−2^ × sr^−1^ × μm^−1^), whereas VIs were computed from reflectance values. We chose the empirical line method (ELM) for atmospheric compensation to convert from radiance to reflectance imagery [40]. This assumes that each pixel is approximately equidistant from the imaging sensor and that illumination is constant across the scene. Our line scanning imager was flown at approximately 100 m and collected an image swath 15 m wide to eliminate concerns about distance, meaning that the center pixel is an average of 29 cm closer than the furthest pixel, or less than a 0.3% difference. The totality of the field imagery was collected in less than 18 min with minimal cloud cover, so we also safely assumed constant illumination over the entire scene. 

With these assumptions satisfied, we were able to use the dark and light Lambertian (diffuse) reflecting panels with flat spectral reflectance values of 6% and 55%, respectively, to obtain and compute our ELM gain and bias factors as outlined by Eismann [40]. We conducted this ELM conversion for the image of each subplot, yielding 36 × 61 × 15 m reflectance images, each with 272 spectral bands. The GSD of this imagery, computed with the altitude *alt*, pixel pitch *p*, and sensor optic focal length *f*, via

(1) $$GSD=\frac{alt\times p}{f}$$

was found to be 0.063 m on average. This high spatial/spectral resolution imagery serves as the basis for our dependency analysis, with one final consideration. 

The Rochester Institute of Technology Chester F. Carlson Center for Imaging Sciences calibration laboratory conducted an analysis of the VNIR imaging sensor, finding that below 420 nm and above 950 nm, sensor effects dominate the noise present in each pixel spectrum due. This is due to keystone, smile, and lens material and structural wavelength dependencies. As such, we removed these bands for consideration in our band selection algorithms. 

### 2.4. Spectral Denoising

Our imaging spectroscopy data had a large noise component, particularly in the blue and NIR bands. Two denoising techniques therefore were investigated to increase the signal-to-noise ratio across all bands: the fast Fourier transform and wavelet denoising. 

First, since the noise present in our reflectance signal had a high frequency across all targets, we investigated denoising through Fourier signal processing. Using the fast Fourier transform (FFT), we masked high frequency data in the frequency domain using a power spectral density (PSD) filter, then performed an inverse FFT (iFFT) back to the spectral domain [41]. This process resulted in sinusoidal behavior, which proved difficult to remove from each subplot spectrum without removing low frequency spectral features of interest, despite removing high frequency noise present in the spectra. For example, the green reflectance peak was suppressed, while the slope of the red edge rise changed significantly. We thus sought to minimize potential feature suppression in subplot spectra, caused by our data pre-processing, when compared to those present in the original data, i.e., features which may be indicative of yield drivers. We opted to use wavelet denoising, an alternative method which has been widely used with imaging spectroscopy data [42].

We leveraged the Bayes Shrink method of wavelet smoothing, using the standard deviation of the noise features identified in each band as the scaling factor and choosing a soft threshold approach. The Bayes Shrink method identifies a threshold for each band, as opposed to applying the same value for the entire spectrum [43]. 

Wavelet denoising was successful at smoothing reflectance spectra of our all six calibration panels and vegetation pixels, while preserving the spectral features of interest which may be correlated with LCC, CC, and other biophysical parameters. It is worth noting that wavelet denoising mitigated, but did not eliminate, the atmospheric absorption feature at the oxygen absorption line (located at 762 nm) and NIR water absorption band at 820 nm [40]. 

### 2.5. Spectral Band Selection

We chose three band selection algorithms, each of which exploits the statistical properties of our imagery in a different manner. These algorithms are Principal Component Analysis—Maximum Variance (PCA-MV), Linearly Constrained Minimum Variance—Constrained Band Selection (LCMV-CBS), and Random Forest (RF) importance scoring. As PCA-MV and LCMV-CBS deal with band statistics only, with no regard for the corresponding yield of an image, RF was chosen to ensure that one method took yield into account when identifying important spectral drivers of the dependent variable. 

The PCA-MV algorithm leverages the principal component transformation using the band covariance matrix of a given image, $\Sigma_{i}$:

(2) $$\Sigma_{i}=x_{i}^{T}x_{i}$$

where $x_{i}$ represents a flattened array of pixels from image *i*.

We then computed the eigenvectors and eigenvalues of this covariance matrix using singular value decomposition. These eigenvalues represent the variance associated with each principal component, while the eigenvector columns contain information about how much each band contributes to the variance represented by the corresponding eigenvalue. To translate these arrays into loading factors for each band *l*, $W_{l}$, we completed a squared sum of each eigenvector along band axis l:

(3) $$W_{l}=\sum_{n=1}^{N}{\left(V_{n,l}\right)}^{2}$$

where $V_{n,l}$ is the lth element of the nth eigenvector and *N* is the total number of eigenvectors [44]. Following this, we compute $\rho_{l}$, the band power index, by multiplying these band weights by their corresponding eigenvectors and normalizing to the sum-total variance i.e., the covariance matrix eigenvalues. These band power indices imply the importance of each band, *l*, as they are derived from the portion of explained variance that the band contributes to the overall image variance.

The second band selection method was LCMV-CBS. Described by Chang and Wang (2006), this method borrows from the principles of the finite response filter to constrain the selection filter response in order to provide not only a ranking of the bands, but also criteria to help determine the number of bands to be selected [45]. One of the benefits of LCMV-CBS over PCA-based dimensional reduction is that the PCA transforms the data into an uncorrelated space, converting the data from its original state and potentially altering the image in an undesirable manner [46].

The LCMV-CBS method uses the set of band images from the original spectroscopic imagery, $B_{l}^{T}$, defining each band image as a single vector containing *NM*-dimensional column vectors (where *M* is the number of columns in the spectroscopic image and *N* is the number of rows), and designing a constrained filter for each band image:

(4) $$B_{l}^{T}v_{l}=1_{N}$$

where $v_{l}$ is an *M*-dimensional column vector of weights which minimizes the output energy of the filter to *N* constraints, and $1_{N}$ is an *N*-dimensional column vector of 1s. This is further defined as an optimization problem:

(5) $$v_{l}=\Sigma^{-1}B_{l}{\left(B_{l}^{T}\Sigma^{-1}B_{l}\right)}^{-1}1_{N}$$

Here, Σ represents the band correlation matrix, which is a square matrix of size *L*, the number of bands:

(6) $$\Sigma =\frac{1}{L}\sum_{l=1}^{L}{B_{l}B}_{l}^{T}$$

Finally, the minimization of the least square error, $\tau_{l}$, represents the correlation that band l shares with the overall image. This factor is defined by the authors as:

(7) $$\tau_{l}=v_{l}^{T}\Sigma v_{l}$$

A larger $\tau_{l}$ value corresponds to a larger ‘importance’ to the overall hyperspectral image due to this higher correlation factor [45]. 

The final band selection method was RF importance scoring, which uses the silage yield as basis for determining which spectral bands are the most important. This method generates importance scores which quantify the success of each band, with the ‘trees’ and ‘branches’ generated as a result of all the individual bands [46]. This method was included in our work because it accounts for the corresponding yield of each subplot and determines the importance of each spectral band by regressing the subplot yield independently, as opposed to the previously mentioned methods, which are canonical and statistical analyses of the imagery, irrespective of yield. 

### 2.6. Spectral Resampling

Spectral resampling was accomplished in radiance space, even though the spectral band selection approach used reflectance to maintain the integrity of the original imagery through the resampling process. The new spectral configurations were based upon the outcomes of each band selection method. Additionally, configurations were made based on bands which were selected by multiple selection algorithms. In total, 11 configurations, plus the original 272-band imagery, were identified. 

These configurations were generated by integrating the original imagery in the radiance domain, based upon the derived band centers and bandwidths. These were determined by assessing adjacent band algorithm outputs and thresholding scores, based on the distance from band center to the noise floor of the algorithm’s output. Finally, a Gaussian resampling function was generated for each new band for every configuration, modeled using the thresholder full-width-at-half-max (FWHM) as the standard deviation for the Gaussian curve. This more closely resembles the relative spectral response typically seen in spectral sensors, as opposed to a rectangle resampling function (which was also computed for comparison). 

**Figure 3:**
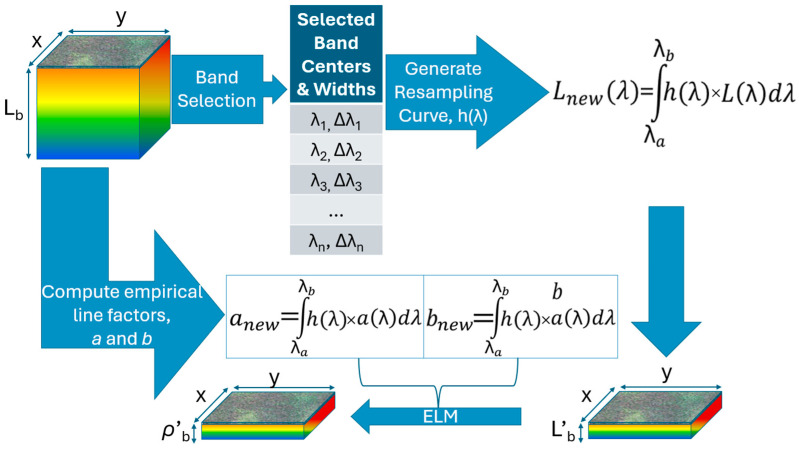
An overview of the spectral resampling process, showing the radiance integration and conversion to the reflectance domain.

The resulting imagery then was converted to the reflectance domain via the same ELM atmospheric compensation method as previously described. The gain and bias terms originally computed for the full spectrum dataset were integrated along the same indices as the spectral data. This is possible due to the linear nature of the ELM reflectance conversion. Figure 3 depicts our spectral resampling process. 

Finally, we resampled the “default” selection results at the thresholded value described above, and then two alternates: narrow and wide. Narrow was defined as 50% the width of the FWHM, while wide was considered 150%.

### 2.7. Spatial Resampling

Spatial resampling was performed to recreate our imagery at multiple GSDs. The new spatial resolutions for our resampled imagery ranged from our original 0.063 m to 30.0 m, or approximately the same GSD as the United States Geological Survey’s (USGS) Landsat system. Our spatial downsampling processing involved tiling our imagery, convolving it with a Gaussian kernel representing the optics of a new imagery collection system, followed by the resampling process to the lower GSD.

The spatial resampling process began with tiling, or repeating, our imagery. This served a dual purpose: first, we wanted to ensure that there were a large enough number of pixels representing each subplot to avoid favoring a dataset with a larger number of features over one with only a few samples. At the original size, the 0.063 m GSD imagery contained approximately 780 k pixels, while the 32.23 m GSD imagery equated to 1–2 pixels. This subplot size also presented a problem for sampling the imagery at the 30 m GSD, because the plots themselves are only 15 m wide, meaning that the new pixels would be sampling at a larger size than the subplots themselves. As a result of the tiling process, the area of each subplot was increased from 0.09 to 0.72 hectares. The yield values for these subplots remained constant but was increased proportionally to account for the increase in area. Secondly this tiling, in addition to zero padding, ensured that our Gaussian convolution did not wrap around pixel values from the right side of each subplot to the left side, thus inappropriately changing the spatial distribution of reflectance values.

We then convolved a Gaussian kernel over the resulting tiled image. This was intended to emulate the optics of a new system, commensurate with the resampled GSD. Typically, these larger GSDs would result from a higher altitude image collection system, like a satellite, and we labored to ensure a realistic representation of this collection scenario. This includes the aggregation that would result from a real collection aperture, which was accomplished with an appropriately sized kernel for the target GSD.

**Figure 4:**
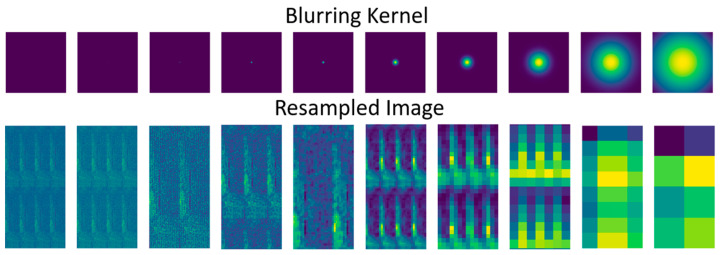
An example of our described resampling process. The top row shows the Gaussian kernel as computed from our aperture size, i.e., the Fourier transform of a new aperture, and the bottom row is the result: a tiled, blurred, resampled image at the new GSD.

Finally, we resampled our tiled, blurred imagery at the new GSD by passing an averaging filter at the selected resolution. This simulated the aggregation for a system which collects at an altitude that would result in the resampled GSD. An example of the tiled imagery and associated resampling can be seen in Figure 4.

Each spectral configuration was spatially resampled at the following nine GSDs: 0.13 m, 0.25 m, 0.5 m, 0.76 m, 2.02 m, 4.03 m, 8.06 m, 16.32 m, and 30m, plus the original 0.063 m GSD, for a total of ten spatial resolutions.

### 2.8. Corn Yield Forecast and Prediction Model

**Figure 5:**
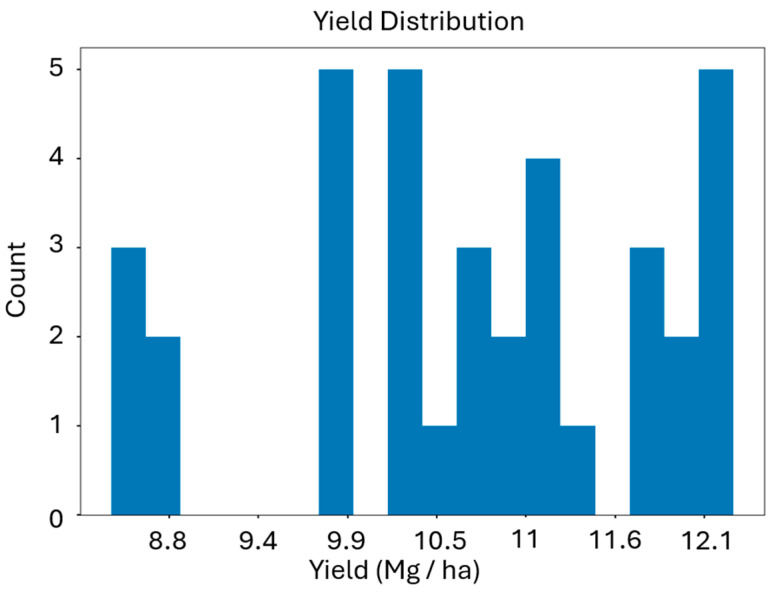
The distribution of yield values within the study area used for this work. Yields are in units of Mg/ha computed for each of the 36 subplots in the field, and we report all yield data in 35% dry matter equivalent. We opted for a non-parametric yield model given that these values do not follow a normal distribution.

As previously discussed, our chosen yield forecast and prediction model was SVR, which defines a decisional hyperplane based upon the input data. This algorithm was chosen because it has been proven to either out-perform or have equal performance to other models at multiple spatial and spectral scales, from three-band point spectrometer reflectance data in Barzin et al. (2022), to MODIS imagery (36-band, 250 m GSD) in Kenduiywo et al. (2014) [14,20,28,47]. Machine learning models have an advantage over linear or exponential regression for data such as ours, which do not follow a normal distribution (Figure 5). This suggests that non-parametric modeling may be more effective than parametric options, as the former rely solely on the data to form the model, as opposed to a statistical representation of the data in the latter case [48].

We next computed the mean spectra for all subplots for each spectral configuration. These mean spectra were used as inputs for our SVR model. We decided to tune the SVR hyperparameters for all 110 unique datasets which result from the spatially and spectrally resampled imagery, as opposed to a single model tuned across all datasets, accomplishing this through use of a parameter grid search by means of leave one out cross validation. This yielded the most robust comparison because all machine learning model hyperparameters were tuned to the training datasets. Our model did not have to be generalized, in fact, it should not be, in order to provide the desired comparison outcome. Because both corn silage and grain yield have shown a nonlinear correlation with spectral reflectance derived VIs, we used the radial basis function (RBF) kernel as it has been shown to accurately model nonlinear effects similar to a multi-layer perceptron machine learning algorithm [29,48].

We used an 80/20 train/test split to divide our data for model development. The split was chosen from an initial random seed and then preserved for all 110 datasets to ensure model performance did not change due to differences in training and test datasets.

### 2.9. Yield Model Performance Metric

Our chosen performance metric for quantifying the performance of yield modeling was mean absolute percentage error (MAPE) as used and described by Liu et al. (2021):

(8) $$MAPE=\frac{1}{N}\sum_{i=1}^{N}\frac{\;\left|y_{test}-y_{predicted}\right|}{y_{test}}$$

where $y_{test}$ is the yield truth data, $y_{predicted}$ is the predicted yield output from our SVR mode, and *N* is the total number of test data points [49]. We also report model performance in terms of root mean square error (RMSE):

(9) $$RMSE=\sqrt{\frac{\sum_{i=1}^{N}{\left(y_{pred}-y_{true}\right)}^{2}}{N}}$$

We report these metrics because Chai and Draxler (2014) showed that both MAPE/MAE and RMSE may reveal more about model performance when combined [50].

## 3. Results

### 3.1. Yield Regressions from PCA-MV Datasets

**Figure 6:**
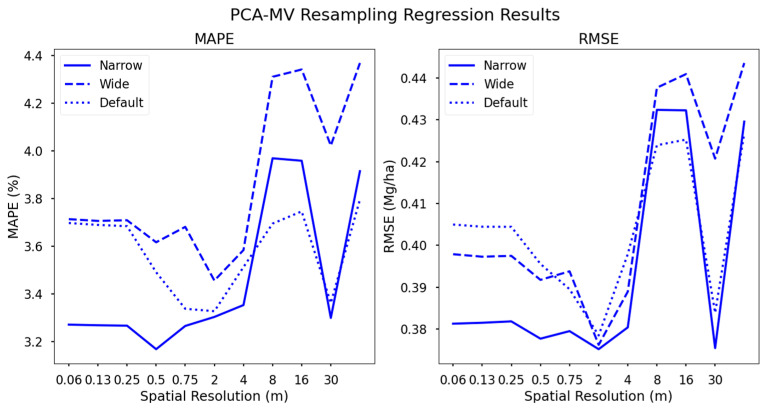
Support vector regression yield estimation results using three variations on PCA-MV derived spectral sampling configurations. Accuracy decreases as spectral complexity decreases. Lower spatial resolutions generally lead to worse performance with the clear exception of the 16 m GSD datasets across all configurations.

The three datasets resulting from the default, wide, and narrow resampling schema described in Section 2.6 performed similarly to one another at some spatial resolutions with respect to RMSE, e.g., 16 m GSD, while there were notable gaps between them at other GSDs (Figure 6). The “narrow” dataset, in particular, outperformed others at most spatial resolutions (excepting 8 and 16 m GSDs), likely in large part due to the increased spectral resolution for that configuration. Our “wide” resampled configuration resulted in six band multispectral imagery, while the default and narrow configurations had seven and 11 spectral bands, respectively. Each contained blue, green, and red visible bands, with a differing number of NIR bands. The narrow configuration contained two blue and two green bands, similar to the Planet SuperDove spectral sampling design [51]. 

Performance changes significantly as GSD increases (Figure 6). Accuracy increases both for MAPE and RMSE at lower spatial resolutions (higher GSDs) until 4 m, for all configurations. This trend was generally applicable to all spectral configurations. We suggest that spatial averaging begins to remove variances present, where such variances are attributable to leaf angle distribution, causing substantial variance in the green and NIR regions, as mentioned above. Furthermore, shadowing due to differences in canopy height is mitigated by this smoothing, as are texture features resulting from the standard deviation of canopy height. While absolute plant height has been shown to be correlated with grain yield, it is difficult to obtain accurate heights from nadir-facing imagery without substantially increasing the number of images collected during an acquisition, thus increasing data storage requirements [31]. 

Data storage requirements of course increase with spectral complexity, which is another factor when considering possible future sensor designs based on these analyses. For instance, our imaging spectroscopy dataset was 65 gigabytes (3.34 ha), while our 12-bit RGB imagery of the same field totaled 1.2 gigabytes. Similarly, our 64-bit default resampled dataset at the highest spatial resolution was 4.5 gigabytes. Quantizing this would further reduce file size, while introducing minimal quantization error.

### 3.2. Yield Regressions from RF Datasets

**Figure 7:**
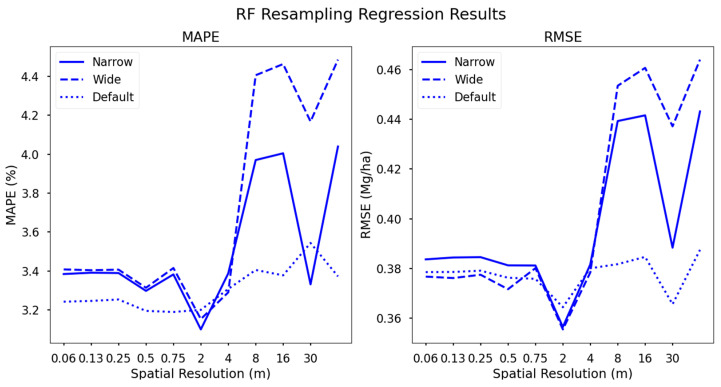
Support vector regression yield forecasting results from the RF importance scoring resampling datasets. The default dataset clearly performs the best across all spatial resolutions with minimal change. The narrow dataset, unsurprisingly, surpasses the dataset likely due to the difference spectral complexity (13 vs. 6 bands).

The datasets resulting from RF importance scoring generally performed best among all spectral configurations. Specifically, the default configuration resulted in a model with the highest accuracy across all spectral configurations, regardless of spatial resolution. The relative difference in performance was small, as seen in Figure 7, but the fact that this configuration continuously out-performed others suggests that it may warrant further investigation in the form of band center and width optimization, taking spectral markers of biochemical components into account. 

We suggest that the narrowband dataset supplies too many features to our SVR model, which may ultimately be uncorrelated with yield, leading to the default recommendations with the most favorable spectral content. This default configuration contains spectral content from the blue, green, red, red edge, along with several NIR bands, while the narrow version contains adjacent bands which are highly correlated with one another. While correlation between adjacent bands may be high, the dimensionality increase from 8–13 bands presents a much more complex hyperplane for the SVR algorithm to optimize.

### 3.3. Yield Regressions from LCMV-CBS Datasets

**Figure 8:**
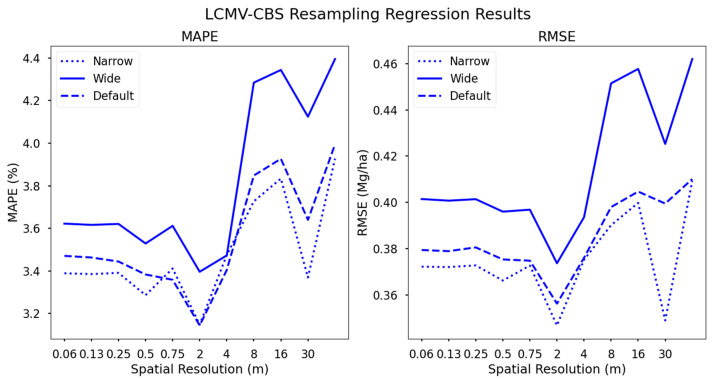
Regression results using our LCMV-CBS dataset. Here, the narrow and default resampled datasets clearly resulted in higher accuracies across all spatial resolutions using a support vector regression yield forecast algorithm.

Our final resampling method, based on LCMV-CBS selections, had similar relative relationships to the PCA-MV dataset between the narrow, wide, and default configurations. These datasets followed the same trend in accuracy as the RF and PCA-MV datasets with respect to spatial resolution, i.e., higher spatial resolutions resulted in lower accuracy (Figure 8).

**Figure 9:**
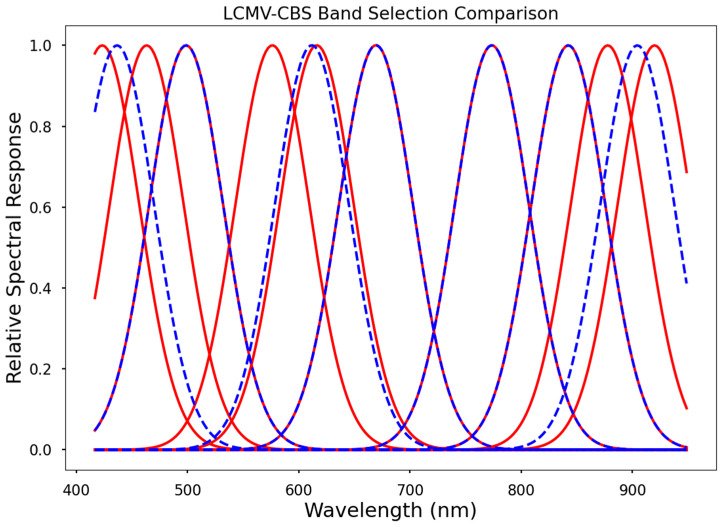
Band selections resulting from the LCMV-CBS default (dotted blue line) and narrow (solid red line) schema as described previously. Note the overlapping selections at 500 nm, 615 nm, 670 nm, 775 nm, and 840 nm, all of which are included in various vegetation indices corresponding to LCC, CC, and other factors correlated with above ground biomass or silage yield [31].

The default and narrow datasets, i.e., seven and ten spectral bands, respectively, performed approximately the same due to high overlap in band centers (Figure 9). It follows that there would be high overlap between narrow, wide, and default datasets, given that LCMV-CBS assesses the correlation of single band images with the hyperspectral image. Generating our narrow and wide datasets is essentially lowering or raising the noise floor of our selection results. It therefore appears that the default selections were the most optimal, retaining important spectral information in the blue, green, red edge, and near-infrared regions, while removing adjacent bands which are highly correlated with one another, as well as bands which are not correlated with biochemical markers. Furthermore, the default configuration contains band centers which are known to be correlated with LCC, CC, and other partner photosynthetic biochemical compounds, as well as NIR bands correlated with vegetation stress and moisture content. It thus follows that including more spectral information results in a more difficult problem for SVR to generate a decisional hyperplane solution. A higher number of features (LCMV-CBS narrow dataset) makes for more decision plane dimensions, while a lower number of features (LCMV-CBS wide dataset). 

### 3.4. Yield Regressions from Overlapping Selections

**Figure 10:**
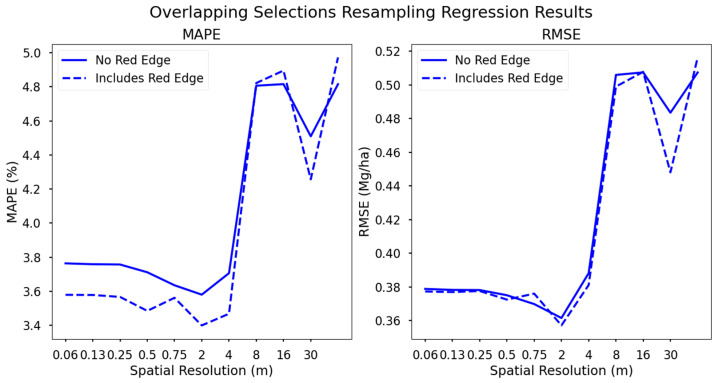
Regression results using the overlapping selections from all band selection algorithms including the red edge (dotted line) or no red edge (solid line). The red edge dataset slightly out-performs the non-red edge dataset. These datasets both have a larger drop in performance beyond 4 m GSD when compared to the other spectral configurations.

Two final datasets were generated which incorporated common band selections in the blue, green, red, red edge, and near infrared regions. This was completed to identify a configuration with low dimensionality but included the highest value spectral content. Support vector regression results from these datasets were comparable to other configurations at higher spatial resolutions but were substantially worse at lower spatial resolutions (Figure 10). 

The difference in performance between these two datasets was attributed to the inclusion of the red edge, centered at 715 nm with a 30 nm FWHM. As previously discussed, the red edge is a spectral reflectance feature in vegetation, the inclusion of which in yield modeling can result in higher accuracies [52]. This comparison serves to evaluate the effectiveness of the red edge in augmenting a typical four-band multispectral sensor (RGB-NIR). 

The dataset which included the red edge did perform slightly better than the dataset without, but the difference was considered marginal. It is likely that the other configurations, which include multiple NIR bands in addition to the red edge, provide a substantial amount of decisional information for SVR to generate an accurate decisional hyperplane for yield forecast regression. 

### 3.5. SVR Regression Results

Our results indicate that in general, the random forest algorithm chose the most appropriate spectral band centers and widths for our purposes, and that results degraded beyond the 2.02 m GSD spatial resolution. Significant degradation in performance occurred beyond 4.03 m GSD, suggesting that the optimal spatial resolution is between 2–8 m GSD. We contend that this is primarily due to favorable mitigation of noise through spatial aggregation, with the added impact of minimizing the abundance of soil spectra in favor of vegetation spectra. This assertion presumes that the SVR model is defining the decisional hyperplane based upon differences in the reflectance spectra of vegetation, which we assume due to the spectral features selected by our dimensionality reduction processes.

**Figure 11:**
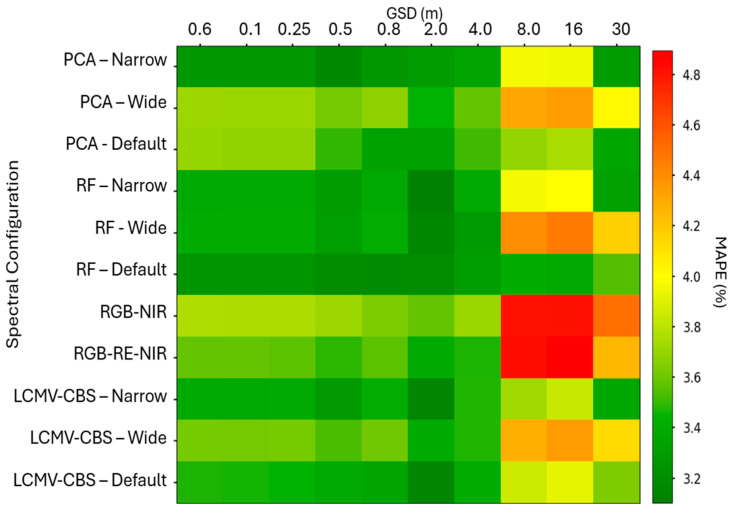
Support Vector Regression yield forecast results from all 110 spectral and spatial combinations. Note the dramatic decrease in performance after 4 m ground sample distance (GSD). The 2 m GSD appears to be the optimal spatial resolution across all spectral configurations. The random forest derived band selections appear to out-perform all others regardless of spatial resolution.

Specifically, the five top performers in terms of MAPE for our yield forecast were the three RF-generated variations and both the original and ‘narrow’ bands resulting from LCMV-CBS, all with 2 m GSD spatial resampling. Figure 11 shows the comprehensive results (MAPE) for all combinations of spatial and spectral resampling, which range from 2.81 to 4.51%.

**Figure 12:**
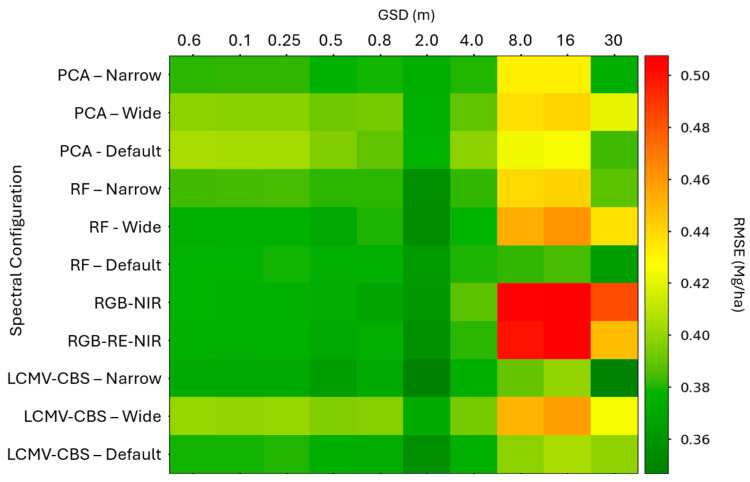
Support vector regression yield forecast results from all 110 spectral and spatial combinations. Note the dramatic decrease in performance after 4 m ground sample distance (GSD). The 2 m GSD appears to be the optimal spatial resolution across all spectral configurations, and the Narrow Linearly Constrained Minimum Variance–Constrained Band Selection configuration appears to slightly out-perform RF selection.

When comparing RMSEs, the relative results were similar but not identical as seen in Figure 12. We still observed a sharp decrease in performance after 4 m GSD, as well as generally optimal performance at 2 m GSD, but the 30 m GSD appeared to exhibit stronger performance when compared to the preceding spatial resolutions. Our RMSE varied from 0.382 to 0.569 Mg/ha. Relating these accuracies to other authors (Table 1), these results are on par with silage estimation from Tunca et al. (2023) and a significant improvement over Aghighi et al. (2020), both of which used Landsat derived NDVI (as opposed to full spectra in our study) for yield regression using linear regression and SVR, respectively. While Aghighi et al. (2020) used time series imagery as SVR model inputs, Tunca et al. (2023) used imagery collected throughout the growing season to assess accuracy as a function of crop maturity [34,37].

Performance trends with respect to spatial resolution were observable with both MAPE and RMSE, but more profound with the RMSE metric. The RF default selection dataset was slightly worse than LCMV-CBS (Narrow). Scoring our combinations with RMSE also revealed that there was a high-performance combination with LCMV-CBS (Narrow) and 30 m GSD. All of these results, however, show that SVR is a promising yield forecasting algorithm at multiple spatial and spectral resolutions.

### 3.6. LCMV-CBS Band Selections

Our LCMV-CVS algorithm selection results strongly favored 475–525 nm and 600–710 nm. Local maximums were also found from 780–815 nm and Figure 8 displays the spectral band ranking resulting from LCMV-CBS. 

**Figure 13:**
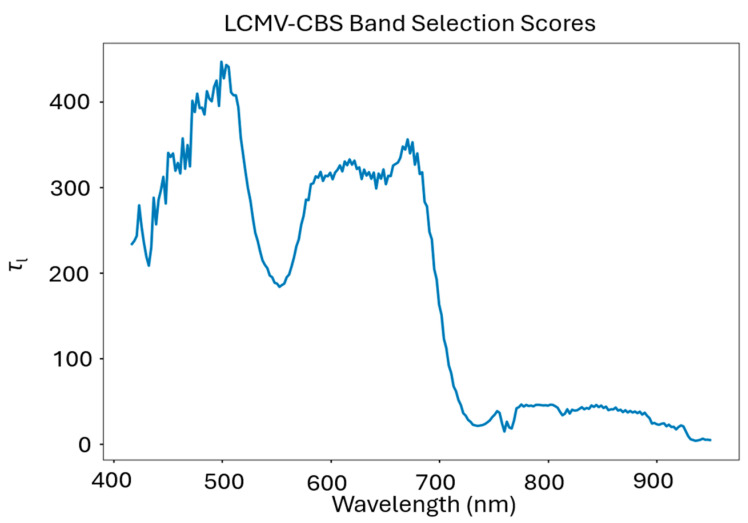
The τ scores from the LCMV-CBS spectral band selection algorithm. These values correspond to the relative correlation that each band has with the overall hyperspectral image, and thus contains more pertinent spectral information.

A notable exception to this were the LCMV-CBS selection results, which de-emphasized the 550 nm region (Figure 13), typically associated with the presence of chlorophyll. This may result from the presence of soil pixels in the imagery (approximately 20%), as these pixels contained a higher reflectance in the red region when compared to the green reflectance in vegetation. 

### 3.7. PCA-MV Band Selections

**Figure 14:**
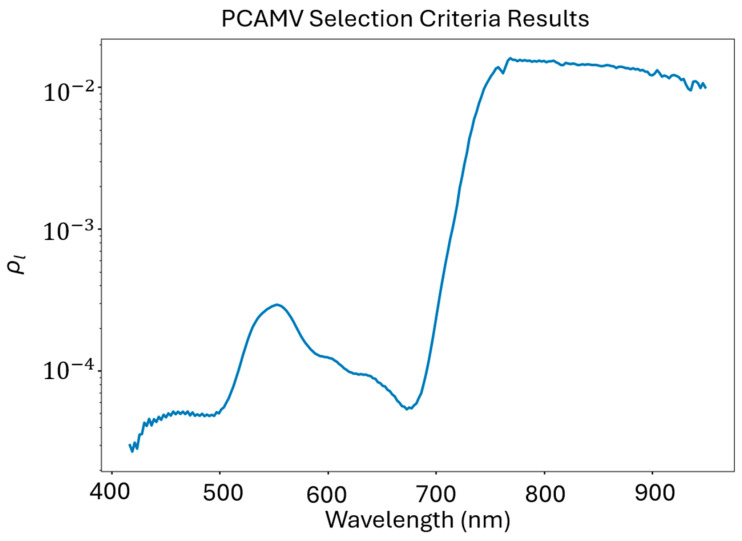
The band power indices computed from our imaging spectroscopy data. These values are the result of performing PCA-MV for all 36 subplot images, totaling approximately 11.34 million pixels.

The PCA-MV algorithm generated band rankings resembling a typical vegetation reflectance spectrum, as seen in Figure 14. This is an expected outcome as the first principal component (PC) normally mirrors the shape of the mean target reflectance spectrum as it accounts for >98% of the variance present in the image. Due to the exponentially decreasing variance explanation of the following principal components, the first PC typically dominates the weight calculation as described in our materials and methods.

### 3.8. Random Forest Importance Band Selections

**Figure 15:**
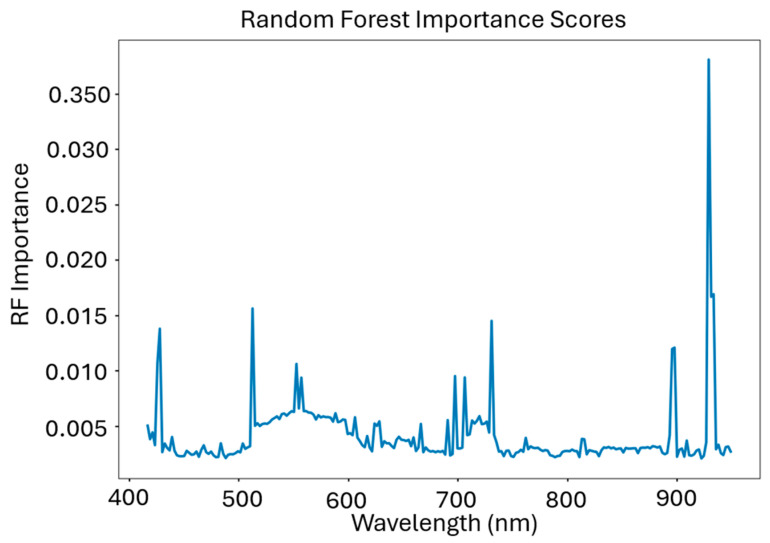
The importance rankings of each band used as inputs for a random forest regressor for the 36 subplot image spectra and their respective yields.

Random forest importance scoring yielded selection results which peak at 430, 550 nm, 670 nm, 725 nm, 815 nm, 890 nm, and 940 nm. Taking the regression-targeted nature of this algorithm into account, these band centers were expected due to known spectral features present in vegetation. Importance scores based upon the RF ranking can be seen in Figure 15.

## 4. Discussion

### 4.1. SVR Yield Regression Sensitivities

There was a slight increase in performance from 8 m GSD to 16 m GSD, after which the decrease continued at 30 m GSD. We observed this trend through multiple different spatial resampling methods before arriving at the schema described above. We contend that this slight increase in performance results from a favorable spatial averaging occurring at this resolution. As previously discussed, spatial averaging can mitigate variances resulting from shadowing, leaf angle distribution variances causing variation in upwelling radiance due to specular reflection from leaves, and other spatial and biophysical features which are not positively correlated with yield. This averaging would also minimize the impact of spectral contributions from soil content at this advanced growth stage (VT) when the canopy has not quite fully closed. 

The most important spectral feature appeared to be centered around the red edge (710 nm), two NIR bands (approximately 820 nm and 920 nm), as well as red, green, and blue bands at approximately 670 nm, 530 nm, and 450 nm respectively. While the exact wavelength centers and related bandwidths varied, these general trends were present in most of the resampled configurations. An intriguing spectral sampling method for the red edge was found to be common among several selection algorithms, each independently sampling on either side of the red edge as opposed to being centered directly on it. We suggest that there is merit to a multispectral sampling of the red edge for this use case, and possibly others, as it avoids the high data dimensionality of imaging spectroscopy while coarsely sampling the red edge rise. This advantage also appears to be further amplified by sampling deeper into the NIR at band centers used for NDVI, EVI, and other VIs shown to have correlation with plant health and above ground biomass. As discussed, the highest SVR yield estimation regression performance lent itself to the RF importance band. 

We performed a grid search hyperparameter optimization using the training data for each dataset to optimize model performance. We found that the regularization parameter consistently had the largest differences between spectral configurations across all spatial resolutions, ranging from 1 × 10^2^ to 1 × 10^4^. This was expected as this parameter is inversely related to the number of features included in each data sample, so datasets which include more features (bands) would have larger regularization parameters when compared to datasets with fewer features [53]. Other hyperparameters did not change substantially, with gamma ranging from 1 × 10^−3^ to 1 × 10^−4^ and all others remaining stable. The relatively tight distribution of these hyperparameters, in combination with the performance trend with respect to spatial resolution, which was generally common to all spectral configurations, implies that SVR is indeed robust to differences in scale. In other words, SVR was deemed to be the preferrable regression technique for field-level yield estimation, as well as regional yield estimation.

The results here also suggest that, regardless of spectral configuration or spatial resolution, SVR presents a viable solution to silage yield regression in addition to the proven grain yield regression performance, as shown by Kenduiywo et al. (2014) and Barzin et al. (2022). This was corroborated by Bhadra et al. (2020), who found that SVR was the top performer in estimating LCC using imaging spectroscopy data when compared to RF, partial least squares regression, and extreme learning regression [14,28,52].

### 4.2. Physical and Biochemical Relationships to Spectral Band Selections

The LCMV-CBS derived correlation of soil pixel spectra with the hyperspectral image increased alongside their abundance, i.e., earlier in the growing season. The relatively minor differences in the reflectance values of the corn leaves, which result from differing levels of biochemicals, leaf angle distribution, and other factors, generate considerable variance in this region, thus decreasing correlation with the hyperspectral image as a whole. This increased variance directly explains the low score with this method in those regions. While machine learning-based yield forecast algorithms may favor spectral features with high variance, it is still important to consider dimensional reduction, in addition to secondary vegetation characteristics. The observation that PCA-MV identified the red spectral region as having relatively high variance also serves as evidence of this hypothesis, along with the same effect occurring in the near-infrared region beyond the red edge. Additionally, dimensional reduction via PCA normally removes a large portion of information which provides little contribution to explained variance. While some PC vectors may be ranked lower in terms of this explained variance, it is crucial to evaluate each for the possibility that they may contribute a key portion of variance.

LCMV-CBS appears to have isolated other spectral features of chlorophyll A/B and similar photosynthetic biochemical markers like phycocyanin, which is reflective in the blue spectral regime [40]. Furthermore, the spectral regions necessary for several important VIs (red, NIR, red edge) scored highly, suggesting that the selection algorithm worked as intended and identified bands of interest. The fact that these bands were correlated with spectral features from imagery with high spectral resolution comes as no surprise, because the local maxima of the scores correspond with spectra features present in vegetation and are associated with CC, LCC, water content, and other biochemical components. We propose these as evidence which may explain the higher performance of these band selections seen with our RMSE results. 

This outcome was anticipated, as the green and NIR regions are the most widely used for biochemical and biophysical spectral correlation studies, e.g., Peng et al. (2012), Cao et al. (2013), and Liu et al. (2021), due to the aforementioned features present in vegetation [2,21,22,49]. We again decided that choosing bands based on the local maxima of these PCA loading-derived scores was most appropriate, setting the FWHM in both the green and red regions based on adjacent values. Particularly in the NIR, we ensured that selected values were not overlapping closer than the FWHM value of the Gaussian resampling curve; this ensured that the majority of the spectral information integrated into the new band was unique.

Our PCA-MV analysis revealed that the entirety of the NIR region contributed heavily to the overall variance of the image, suggesting that water content, leaf area index, and leaf angle distribution may be major contributors to this variation. The selection of appropriate bands in this region carefully considered the most likely source of spectroscopic image variance, avoiding the possibilities that the observed variance wasn’t derived from atmospheric (scattering) sources and included solely for that reason. The variance contribution of other objects and materials present in the image, like irrigation or farming equipment, should be retained, as these may contribute to the success of machine learning models for CC, LCC, and yield forecasting and prediction.

Both LCMV-CBS and PCA-MV identified the oxygen absorption band (~761 nm) and atmospheric water absorption bands (~823 and 930–950 nm), ranking these atmospheric transmission features low relative to their neighboring bands, further signifying the importance of local maxima as opposed to global maxima for band selection algorithm ranking. Additionally, knowledge of these transmission spectral features is imperative when performing spectral band selection methods, which may not capture their omnipresent nature. PCA-MV and LCMV-CBS identify such absorption features through different mechanisms. For PCA-MV, the absorption features were present in every pixel, thus they contributed little variance to the image. These features are relatively small when bearing in mind the operation of LCMV-CBS, i.e., they occur over fewer spectral bands, compared to the absorption spectra of the vegetation and other materials present, so they are not necessarily correlated with the entirety of the imaging spectroscopy data.

There were several bands selected by RF importance ranking which overlap with the other two selection methods in the green, red edge, and NIR regions in particular, despite the more abstract appearance of these selections. There was a peak at approximately 428 nm, which corresponds to absorption features in phycocyanin and xanthophyll, indicating that secondary photosynthesis biochemicals may be important contributors for machine learning applications involving the remote sensing of vegetation [49]. Additionally, there were high importance scores for all bands between 510–600 nm, with spikes located at 512 nm and 555 nm. These regions correspond to the chlorophyll absorption spectrum and reflect band selections in PCA-MV, but not LCMV-CBS, which de-emphasized the green region [40].

The red edge (700–720 nm) also featured strongly in importance ranking, as did several other regions in the NIR region, particularly 815 nm, 895 nm, and 925 nm. These NIR bands correspond to several VIs found in literature, e.g., the Modified Chlorophyll Absorption Reflectance Index, Modified Triangular Vegetation Index, Normalized Difference Water Index, and Normalized Difference Chlorophyll Index, thereby suggesting that our RF algorithm is independently leveraging spectral information tied to biochemical and biophysical absorption features [54,55,56].

### 4.3. Revisiting Selection Scheme and Resampling Methodology

We also analyzed a derivative (shape) analysis variant using the first derivative of each pixel, in addition to the reflectance spectra inputs to each of our spectral selection algorithms. Both PCA-MV and LCMV-CBS selected the oxygen and water absorption bands as high scoring candidates, due to the distinctive features in the reflectance domain at these wavelengths. The first derivative did exhibit importance in the case of both the PCA and LCMV-CBS selection algorithms, which identified the red edge as an important feature due to the sharp rise in reflectance. Interestingly, this was not necessarily a high scoring region when those methods were provided with the original spectra. Band selection using this approach was not as informative as the standard reflectance, given especially the emphasis that PCA-MV and LCMV-CBS place on atmospheric features. Masking these features still resulted in a de-emphasis of known regions of interest, though further investigation is warranted into the possibility of determining LCC and CC from these metrics. 

We also investigated a rectangular resampling function, as opposed to the Gaussian resampling strategy employed in this work. We discovered, however, that this approach generally led to 25–50% lower performance when submitted as inputs for the SVR yield forecast when comparing identical spectral band selection results and spatial resolution combinations. We attributed this drop in performance to the truncation of spectral content resulting from the rectangle function, when compared to the Gaussian resampling function which includes weighting of peripheral bands around the center. Clearly the absence of these data in the final reflectance product, after ELM conversion, plays a role in defining the optimal decision hyperplane with SVR, despite the fact that this content is integrated into a single band.

### 4.4. Future Work

Future work should investigate if this method can be used to train an SVR corn silage yield model on data from one year, to test on another. Furthermore, because previous corn silage yield regression models have been shown to be dependent upon geographic location due to varying climate, soil, and other factors, the dependency on these location-specific factors should be investigated. The temporal evolution of yield forecast accuracy throughout the growing season should also be investigated, as the dependable interpretation of model outcomes may be used by farmers to consult management decisions. To counter overfitting concerns, 

Additionally, an examination into a multi-year yield estimation model should be accomplished using an optimal sensor configuration. Furthermore, using our resampling techniques on a high spatial and high spectral resolution dataset to match known systems, e.g., Landsat, MODIS, or Planetlabs SuperDove may be beneficial to compare results with a hypothetical sensor parameterized by this analysis.

Finally, an investigation into the drivers of yield resolvable by remote sensing modalities like LCC, carotenoid content, moisture content, planting density, plant height, and the spectral and spatial dependencies thereof is warranted. These factors are all correlated with yield, and VIs have been created to model their abundance in vegetation from imagery data. As demonstrated, the values of these indices can change depending on the band centers and widths of the sensors used to gather the data. Without a ubiquitous understanding of the impact of these sensor parameters on these biochemical and biophysical characteristics, generalized yield estimation models will be of varying dependence, with unknown weight being given to sensor and data collection configuration. 

Analysis of real data is cumbersome due to ground truth data collection restraints. Typically, to gather truth data for an experiment with similar methods as presented here, destructive sampling must be accomplished to ascertain LCC, CC, and other biochemical abundance [26]. This leads to undersampling of the field, leading to small amounts of truth data relative to the remote sensing data collected. To overcome this, we propose a future study which generates synthetic imagery using PROSPECT and SAIL vegetation reflectance property modeling combined with high-fidelity structural modeling of a corn plant throughout the growth cycle be used to generate large amounts of high-confidence truth data. 

## 5. Conclusions

We conclude that a seven-band, multispectral imaging sensor, with band centers and bandwidths listed in Table 1, are most advantageous for use in SVR-based yield estimation modeling. The SVR algorithm, previously shown to exhibit favorable performance at multiple spatial resolutions when compared to RF, gradient boost, and other ML regression methods, provided relatively consistent responses across the GSDs tested [14,28,52]. This configuration achieved higher accuracy than other spectral sampling configurations in 90% of regression trials (see Figure 11).

This spectral configuration, identified by RF importance scoring, takes into account the red edge through indirect sampling. It samples before the red edge rises at 686 nm, and then after the rise at 766 nm. The width of the post-rise sample band avoids the problem of oxygen absorption near that wavelength through a wider bandwidth, which is relevant since the atmospheric oxygen absorption feature is spectrally narrow. Sampling this pivotal spectral feature in this manner reveals the extent of the red edge rise, which is closely related to health and stress in vegetation as well as nitrogen content [57]. 

Accuracy decreases inversely proportional to spatial resolution, with a notable performance loss after 4 m GSD across all spectral configurations. This relative decrease in performance is comparable to the performance reported in literature, even at the lowest accuracies. We thus concluded that, in general, SVR is robust to scale and could be considered the preferred regression algorithm for yield estimation at the field-scale and regional scale when using remote sensing data as the only input.

These results suggest an SVR-based corn silage yield estimation model is feasible for both grain and silage yield and that such a model would be best utilized with data from a sensor which is designed with the spectral sampling parameters found in Table 2.

## Figures and Tables

**Table 1:** Corn silage yield regression results comparison.

Author	Regression Model	Data Source	RMSE
Tunca	Linear Regression	Landsat-8 (4 plots)	0.29 Mg/ha
Aghighi	SVR	Sentinel-2/Landsat-8 (40 plots)	6.03 Mg/ha
Our Methods	SVR	UAV-Mounted Imaging Spectrometer (36 plots)	0.38 Mg/ha

**Table 2:** Spectral band centers and widths resulting from RF importance ranking.

Band Number	Band Center (nm)	Nominal Bandwidth (nm)
**1**	**432**	**30**
**2**	512	30
**3**	**592**	**30**
**4**	686	20
**5**	**766**	**30**
**6**	844	20
**7**	**929**	**10**

## Data Availability

The data used in this research are not available in order to protect the privacy of the farmers.
